# Progress of rituximab in the treatment of systemic lupus erythematosus and lupus nephritis

**DOI:** 10.3389/fmed.2024.1472019

**Published:** 2024-10-04

**Authors:** Shouqi Mo, Yilan Li, Junbing He, Ling Lin

**Affiliations:** ^1^Department of Rheumatology and Immunology, The First Affiliated Hospital of Shantou University Medical College, Shantou, Guangdong, China; ^2^Department of Rheumatology, Jieyang People's Hospital, Jieyang, China; ^3^Department of General Family Medicine, Jieyang People's Hospital, Jieyang, China; ^4^Jieyang Medical Research Center, Jieyang People's Hospital, Jieyang, China; ^5^Department of Rheumatology, Shantou University Medical College, Shantou, China

**Keywords:** rituximab, systemic lupus erythematosus, lupus nephritis, B cells, treatment

## Abstract

Systemic lupus erythematosus (SLE) is a chronic autoimmune disease with heterogeneous clinical manifestations, often leading to significant morbidity and mortality, particularly due to lupus nephritis (LN). The standard therapeutic approach involving mycophenolate mofetil, cyclophosphamide, and glucocorticoids has shown limitations due to cumulative toxicity and side effects. The introduction of biologic agents, especially rituximab (RTX), a chimeric monoclonal antibody targeting CD20+ B cells, has revolutionized the treatment landscape. This review synthesized the current understanding of B cells’ role in SLE and LN and evaluates RTX’s therapeutic impact. B cells contribute to disease pathogenesis through autoantibody production and immune complex formation, leading to tissue damage. RTX’s mechanisms of action, including Complement-Dependent cytotoxicity (CDC), antibody-dependent cell-mediated cytotoxicity (ADCC), and induction of apoptosis, have demonstrated efficacy in both SLE and LN treatment. Clinical studies have reported remission rates and improved renal outcomes with RTX use, although challenges such as human anti-chimeric antibody development and optimal dosing persist. The review emphasized the need for continued research to elucidate RTX’s long-term benefits and risks, and to explore personalized treatment strategies that incorporate B cell biology for better disease management in SLE and LN.

## Introduction

Systemic lupus erythematosus (SLE) is a chronic autoimmune inflammatory disease characterized by a recurrent-remission course and a broad spectrum of clinical manifestations (1, 2). Globally, the incidence and prevalence of SLE vary significantly by geographical location, with the highest rates observed in North America, while Africa and Australia report the lowest. Factors such as age, gender, and ethnicity play crucial roles in determining clinical outcomes and disease management. Notably, SLE is more prevalent among females; however, disease progression tends to be more severe and rapid in males, resulting in a poorer prognosis. These differences may be attributed to varying environmental factors and genomic differences (3).

To date, the pathogenesis of SLE remains unclear. It can be associated with the interplay of genetic susceptibility, environmental factors, immune system irregularities, and hormonal influences (4, 5). In pathological conditions, patients with SLE typically produce a large number of antibodies, leading to the formation of antigen–antibody complexes. These complexes deposit in the kidneys, causing renal damage, and ultimately resulting in lupus nephritis (LN) (6, 7). Consequently, LN is a severe complication and one of the most common clinical manifestations of SLE, as well as one of the leading causes of mortality among SLE patients. Approximately 60% of individuals with SLE may develop LN, and 5 ~ 20% of those with LN progress to renal failure within 10 years (8–12). Therefore, the primary goals of LN treatment are to control disease activity, prevent relapses and progression, and avert the development of end-stage renal disease.

First-line treatments for LN typically include mycophenolate mofetil (MMF) or cyclophosphamide (CYC) combined with glucocorticoids (GC). Maintenance therapy typically involves MMF or azathioprine (AZA) combined with low-dose GC. The remission rate after 1 year of LN treatment ranges from 30.4 to 66.2%, with a good renal response being associated with improved disease prognosis (13, 14). Despite the introduction of emerging immunosuppressants such as tacrolimus (TAC) and MMF, the complex pathophysiological characteristics of SLE and LN and the clinical difficulty of controlling the disease with a single drug pose significant challenges to treatment. These challenges are further compounded by the notable side effects and cumulative toxicity of immunosuppressive drugs, including ovarian failure, bone marrow suppression, gastrointestinal symptoms, teratogenicity, and an increased risk of malignancies (15). Glucocorticoids (GC) are the cornerstone of SLE and LN treatment. However, prolonged use can lead to various serious long-term adverse effects and an increased risk of infections. It is also associated with an elevated risk of early cardiovascular disease, with SLE patients experiencing a 2- to 4-fold increase in the risk of coronary artery events (16). Studies have indicated that cumulative GC doses are significantly associated with an increased risk of cataracts and osteoporosis with fractures. Additionally, prolonged GC use elevates the risk of ischemic necrosis, diabetes, and hypertension (17). Furthermore, the current treatment regimens for SLE and LN remain limited in their efficacy, with up to 28% of patients eventually progressing to end-stage renal disease (ESRD) or death (18). Therefore, additional therapeutic strategies are needed to improve the prognosis of LN patients. Given the crucial role of B cells in the development and progression of dysregulated immune responses, the use of B cell-depleting agents in the treatment of SLE and LN remains a topic of ongoing debate. Rituximab (RTX), an anti-CD20 monoclonal antibody (mAb), has been extensively used in SLE and other rheumatologic disorders. Thus, we synthesized the current understanding of the role of B cells in SLE and LN, and further summarized the progress of RTX in the treatment of SLE and LN.

## Role of B cells in the pathogenesis and progression of SLE and LN

In patients with SLE and LN, B cells play a multifaceted pathogenic role, characterized by abnormalities in their differentiation and function. B cells contribute to immune damage through multiple mechanisms, including the production of autoantibodies, which can induce immune injury via antibody-dependent cell-mediated cytotoxicity (ADCC) or complement-dependent cytotoxicity (CDC). Additionally, B cells play a crucial role by producing various cytokines, presenting antigens, regulating immune responses, and providing co-stimulatory signals, highlighting their significant involvement in the pathogenesis of SLE and LN (19, 20). As a B cell hyperactivity-driven, non-organ-specific autoimmune disease, SLE is characterized by several B cell abnormalities. These abnormalities primarily function to promote the production of autoantibodies (21). Autoimmune dysregulation leading to the production of autoantibodies against various cellular components is a hallmark of the disease, particularly against nuclear antigens. Over 95% of patients possess autoantibodies targeting antinuclear antigens (antinuclear antibody, ANA) (22). Autoantibodies, as the core pathological feature of the disease, include characteristic antibodies against double-stranded DNA (dsDNA), histones, and the entire nucleosome, as well as antibodies against RNA-binding proteins. These proteins notably include Sm, U1RNP, Ro (SS-A), La (SS-B), and hnRNP A2 (RA33), along with phospholipids (e.g., cardiolipin) or phospholipid-binding proteins like *β*-2-glycoprotein-I. Under normal circumstances, these charged antibodies tend to form immune complexes to some extent, which deposit in the glomerular basement membrane (GBM) of the kidneys, thereby progressing to LN (23). As early as 1967, dsDNA antibodies were discovered in renal tissue biopsies from LN patients, and high specificity for SLE (24, 25). Moreover, the reciprocal changes in elevated dsDNA antibodies and serum complement levels (C3 and C4) serves as markers for onset, classification, diagnosis, and disease activity assessment in SLE and LN. These levels also correlate with treatment response, making autoantibodies valuable as therapeutic diagnostic biomarkers for outcome measurement in routine clinical practice and clinical trials (26, 27). Advancements in technology have enabled more sophisticated and precise analyses of autoantibodies, providing new insights into SLE pathogenesis and positioning it at the forefront of autoimmune mechanism research.

Self-reactive B cells, a type of lymphocyte, produce autoantibodies leading to autoimmune diseases. The mechanisms behind the generation of self-reactive B cells remain unclear, but potential mechanisms include: (1) During B cell development in the bone marrow, abnormalities in the central checkpoint may lead to a vast diversity in the pre-B cell receptor repertoire. This results in the failure of apoptosis of self-reactive B cells, allowing their survival; (2) T cell-dependent B cells, upon antigen and T cell stimulation, enter the germinal center. During the process of somatic hypermutation, abnormalities in negative selection can result in the production of self-reactive B cells (28). Under normal conditions, the survival and activation of self-reactive B cells in the body are regulated by multiple checkpoints. The immune system effectively modulates self-reactive B cells through negative selection mechanisms, including clonal deletion, clonal anergy, and receptor editing, which suppress their proliferation and promote B cell immune tolerance. Consequently, the development of self-reactive B cells is effectively inhibited.

However, when central and peripheral checkpoints become dysfunctional due to factors such as abnormal levels of B lymphocyte stimulatory factors, defects in inhibitory receptors on self-reactive B cells, lowered activation thresholds for B cells, impaired clearance of apoptotic products, and genetic abnormalities, self-reactive B cells can become activated and proliferate. This breakdown in B cell self-tolerance leads to the production of autoantibodies against self-antigens, resulting in the onset of autoimmune diseases such as SLE and LN (29, 30). Yurasov et al. discovered that in patients with SLE, the number of self-reactive mature naive B cells was double that of healthy individuals. This increase was accompanied by a higher quantity of polyreactive antibodies (30). Additionally, high levels of B lymphocyte activating factor (BAFF) from the tumor necrosis factor family and type I interferons (IFNs) may enhance the survival of self-reactive B cells (31). Therefore, B cell abnormalities, particularly self-reactive B cells, play a crucial role in the pathogenesis of SLE and LN. Given the role of B cells in the development of dysregulated immune responses, B cell depletion therapy has been pioneered in patients with refractory SLE and LN.

## B cell-targeted therapy

To date, targeted therapies against autoreactive B cells have become significant therapeutic strategies for systemic lupus erythematosus and various other autoimmune diseases, such as rheumatoid arthritis, multiple sclerosis, and autoimmune thrombocytopenia. The mechanisms of B cell-targeted therapies can be broadly categorized into the following strategies: elimination of autoreactive B cells, blockade of extracellular soluble factors or receptors, intrinsic blockade of B cell activation pathways, and receptor editing (32). Numerous emerging agents are currently under development based on these mechanisms.

In terms of eliminating autoreactive B cells, the predominant approaches involve monoclonal antibodies and CAR-T cell therapy. Monoclonal antibodies target various CD molecules expressed at different stages of B cell development and maturation. These antibodies mediate B cell elimination through antibody-dependent cellular cytotoxicity (ADCC) and complement-dependent cytotoxicity. Examples include anti-CD19 antibodies such as inebilizumab, obexelimab, and tafasitamab; anti-CD20 antibodies like rituximab, ocrelizumab, and obinutuzumab; anti-CD22 antibody epratuzumab; and anti-CD38 antibody daratumumab. CAR-T cell therapy involves the genetic modification of T cells to express chimeric antigen receptors (CARs) that recognize B cell surface molecules, thereby leveraging T cell-mediated cytotoxicity for effective B cell elimination (33). Examples include Anti-CD19 CAR-T cells and BCMA-CD19 compound CAR-T cells.

Regarding the blockade of extracellular soluble factors or receptors, recent research has focused on agents that inhibit BAFF (B-cell activating factor). BAFF, a member of the TNF superfamily, plays a critical role in preventing B cell apoptosis and promoting B cell differentiation (34). Relevant therapeutic agents include belimumab, tabalumab, blisibimod, and ianalumab. Telitacicept is a dual-target agent that inhibits both BAFF and APRIL, with analogous mechanism agents including atacicept and povetacicept.

The elevated expression of type I interferons is associated with both innate and adaptive immune dysfunctions in systemic lupus erythematosus (35). For instance, anifrolumab, a type I IFN receptor inhibitor. Other agents targeting this pathway include rontalizumab and sifalimumab.

Beyond BAFF and IFN-*α*, other cytokine-targeted therapeutics are under development, such as IL-12 and IL-23 inhibitors like ustekinumab and IL-6/sIL-6R inhibitors including sirukumab, tocilizumab, and vobarilizumab (a soluble IL-6 receptor). CD40 ligand inhibitors, such as dapirolizumab pegol, are also being explored.

For the intrinsic blockade of B cell activation pathways, relevant drugs include SYK inhibitors (cevidoplenib, lanraplenib), BTK inhibitors (rilzabrutinib, fenebrutinib, Evobrutinib), and proteasome inhibitors (bortezomib). In the domain of receptor editing, research is ongoing to use edited CAR-Treg cells to suppress autoreactive B cell functions. Additionally, theoretically, receptor editing of autoreactive B cells could potentially avoid autoreactive binding, though no such therapeutics have yet been developed, which might be a future exploration direction.

## Mechanism of action of RTX in B cell-targeted therapy

The anti-CD20 monoclonal antibody RTX is the first to receive FDA approval for the treatment of CD20-positive B cell malignancies, such as non-Hodgkin lymphoma and chronic lymphocytic leukemia. Subsequently, its therapeutic reach has expanded to autoimmune diseases, including rheumatoid arthritis (RA), SLE, and LN (36–38). The therapeutic effectiveness of rituximab is based on its impact on B cells. Consequently, the recognition that B cells play a more important part in autoimmune diseases than last believed has resulted in its growing usage for off-label reasons. The multifaceted mechanisms by which RTX treats SLE and LN include: (1) Complement-Dependent Cytotoxicity (CDC): RTX effectively binds to C1q, activating complement *in vitro*, leading to a cascade reaction and the formation of a membrane attack complex, inducing lysis of CD20+ B cells; (2) Inhibition of Cell Proliferation and Induction of Apoptosis: RTX cross-linking by cells expressing Fc receptors induces apoptosis through the activation of the Caspase-3 signaling pathway, leading to B lymphocyte apoptosis. Additionally, RTX can directly induce cell death via Fab-mediated pathways; (3) Antibody-Dependent Cell-Mediated Cytotoxicity (ADCC): RTX induces the aggregation of monocytes, macrophages, and natural killer cells through the binding of their Fc receptors to the Fc fragment of RTX, leading to the lysis of B lymphocytes (39). Although ADCC is identified as the primary action mechanism of RTX, complement-mediated cytotoxicity also plays a significant role; (4) Follicular regulatory T (Tfr) cells are a specific subset of regulatory T cells concentrated mostly in the germinal center (GC), which act as regulators of GC responses. They may interfere with the identification mechanism of Tfh cells and B cells, trigger Tfh death, and inhibit B cell activity. Using RTX to rebuild GC responses might also contribute to the treatment of SLE (40). Additionally, RTX can affect the function and number of T cells, further contributing to its therapeutic profile. B lymphocytes may function as antigen presentation cells (APC) for T lymphocytes, resulting in a pro-inflammatory response via generating cytokines.

## Progress in RTX drug research for SLE and LN treatment

The clinical studies that have been published regarding the treatment of SLE and LN related to RTX are shown in Table 1 (41–68).

**Table 1:** Published clinical studies on the treatment of SLE and LN related to RTX.

	Country	Study design	Cases(N)	RTX DOSE	Other drugs use	follow-up time(Month)	Outcome
Sfikakis et al. (41)	Greece	PCS	10	375 mg/m^2^ *4	P	12	CR:50% TR:80%
Vigna-Perez et al. (42)	Mexico	PCS	22	2 × 0.5–1 g	NM	3	CR: 23% TR: 55%
Gunnarsson et al. (43)	Sweden	PCS	7	375 mg/m^2^ *4	CYC: 2 × 0.5 mg/m2, MTP: 4 × 100–250 mg, P	6	CR: 43% TR: 86%
Lindholm et al. (44)	Sweden	RCS	17	375 mg/m^2^ *4	NM	12	CR: 12% TR: 65%
Boletis et al. (45)	Greece	PCS	10	375 mg/m^2^ *4	MMF: 2 g/d, P	38	CR: 70% TR: 80%
Melander et al. (46)	United Kingdom	RCS	20	375 mg/m^2^ *4	NM(CYC 3pts)	22	CR: 35% TR: 60%
Pepper et al. (47)	United Kingdom	PCS	18	2*1 g	MMF: 1 g/d, MTP: 2 × 500 mg, P	12	CR: 33% TR: 67%
Garcia-carrasco et al. (48)	Mexico	RCS	13	2*1 g	MTP: 2 × 500 mg	6	CR: 38% TR: 76%
Ramos-casals et al. (49)	Spain	RCS	49	375 mg/m^2^ *4 or 2*1 g	NM	26	CR: 80%
Catapano et al. (50)	United Kingdom	RCS	11	4 × 375 mg/m2 (4pts) 2 × 1 g (7pts)	CYC: 500 mg MTP: 500–1,000 mg	4	CR: 36% TR: 91%
Jónsdóttir et al. (51)	Sweden	PCS	25	375 mg/m^2^ *4	CYC: 2 × 0.5 g, MMF (2pts), P	12	CR: 16% TR: 56% (6 m) CR: 20% TR: 80% (12 m)
Davies et al. (52)	United Kingdom	PCS	18	2*1 g	CYC: 2 × 0.5 g, MTP: 2 × 500 mg	6	CR: 61% TR: 72%
Condon et al. (53)	United Kingdom	PCS	50	2*1 g	MTP: 2 × 500 mg, MMF: 0.5–1.5 g/d	12	CR: 52% TR: 86%
Tsanyan et al. (54)	Russia	PCS	45	1*0.5 g (2pts) 2*0.5 g (16pts) 3*0.5 g (1pts) 4*0.5 g (13pts) 1*1 g (3pts) 2*1 g (11pts)	MTP: 6*250-1000 mg	6	CR: 81% TR: 86%
Contis et al. (55)	France	RCS	17	4*375 mg/m2 (10pts) 2*1 g (7pts)	MTP: 100–750 mg	12	CR: 24% TR: 53%
Kotagiri et al. (56)	Australia	PCS	14	1*375 mg/m2	AZA (6pts), MMF (7pts), CYC(1pts)	6	CR: 14% TR: 79%
Chavarot et al. (57)	France	RCS	15	4*375 mg/m2 (6pts) 2*1 g (9pts)	P	6	CR: 27% TR: 80% (6 m) CR: 47% TR: 60% (12 m)
Hogan et al. (58)	France	RCS	12	2*1 g	MTP: 500 mg, MMF: 1200 mg/m^2^/d	6	CR: 75% TR: 100% (6 m) CR: 75% TR: 100% (12 m)
Rovin (59)	Latin America	RCT	144	1 g*4d	NM	12	CR:34.7% TR: 59.7%
Zhang et al. (60)	China	RCT	84	375 mg/m2 *4	CTX:800 mg/m, P	12	CR:64.3% TR: 83.3%
Moroni et al. (61)	Italy	RCS	37	1 g*2	CYC:1–2 mg/kg/d,MYC:1–2 g/d or AZA:1–2 mg/kg/d,P	12	CR:70.6% TR: 100%
Goswami et al. (62)	India	RCS	222	1.9 + 0.25 g	LDCyC:500 mg/2 week, HDCyC:750–1,200 mg/m, MMF:1.5–3 g/d	6	CR:72.7% TR: 81.8%
Roccatello et al. (63)	Italy	RCS	60	375 mg/m^2^ *8	MMF:2 ~ 3 g /d, CYC: 500 mg, a total of 3,000 mg	12	CR:93.3% TR: 100%
Moroni et al. (64)	Italy	RCS	24	2 g	MMF:1.5–3.0 g/d, P	12	CR:26.4% TR:56.9%
Tanaka Y et al. (65)	Japan	RCS	115	375 mg/m^2^ *4 OR 1 g/m^2^ *1 ~ 2	HCQ:192.3 ± 144.1(13pts); MMF:963.0 ± 921.7 (27pts); TAC:2.1 ± 1.5 (23pts); AZA:57.3 ± 66.5(9pts); MZR:134.9 ± 109.3(8pts); CYC:124.0 ± 121.0(5pts); MTX:11.0 ± 1.4(week) (2pts), P	NM	CR: 20.8% TR: 52.5%
Tanaka et al. (66)	Japan	RCS	34	1 g*4	P	13	CR:37.65% TR: 32.2%
Iaccarino et al. (67)	Italy	RCS	134	1 g*2(118pts) 375 mg/m^2^ *(27pts)	CYC:750 mg*2, P	12	CR:57.8% TR: 84.4%
Condon et al. (53)	United Kingdom	RCS	50	1 g*2	MMF:500 mg*2, MPA:1.2–2.4 mg/L, P	13	CR:52% TR: 86%
Davies et al. (52)	United Kingdom	RCS	18	1 g*2	CYC:500 mg, P	6	CR:61.1% TR: 72.2%
Li et al. (68)	China	RCS	19	1 g	CTX:750 mg, P	12	CR:21.1% TR: 78.9%

AZA, Azathioprine; CR, Complete remission; CS, Control study; CYC, Cyclophosphamide; d, Day; HDCyC, High-dose cyclophosphamide; LDCyC, Low-dose cyclophosphamide; m, Month; MMF, Mycophenolate mofetil; N, The number of patients with available data for analysis; NM, Not mentioned; P, Glucocorticoids; PR, Partial remission; RCS, Retrospective case series; RCT, Randomized controlled trial; RTX, Rituximab; TR, Total remission.

## Clinical usage and dosage of RTX

In August 1997, the chimeric mouse/human monoclonal antibody (mAb) RTX, which targets the B cell CD20 receptor, was approved for use in follicular lymphoma. Edwards et al. were pioneers in demonstrating its effectiveness and safety in the treatment of rheumatoid arthritis (RA) (36). In a randomized, double-blind, controlled study involving patients with active rheumatoid arthritis (RA) receiving methotrexate (MTX) treatment, a single course of two infusions of RTX significantly improved disease symptoms at both week 24 and week 48 compared to MTX alone or in combination with cyclophosphamide (CYC) or continuous MTX therapy (37). Additionally, Leandro et al. were the first to publish an open-label study involving six female patients with refractory SLE who were resistant to standard immunosuppressive therapy. This study provided preliminary evidence for the safety and efficacy of RTX in the treatment of refractory SLE (38). To date, the clinical usage and dosing of RTX vary by condition. For lymphoma and pediatric autoimmune diseases, the standard dosage is 375 mg/m3 for 4 weeks. For conditions such as SLE and RA, the dosage often increases to 100 mg administered twice over 2 weeks. The dosing of RTX for treating SLE and LN typically falls between these two regimens (69). However, a multicenter systematic review involving 1,370 patients with systemic autoimmune diseases treated with biologics found that rituximab (RTX) treatment for refractory SLE might be more effective when using the lymphoma treatment regimen (375 mg/m3 for 4 weeks) compared to the two-week doses of two 100 mg (70). However, based solely on the aforementioned review, it is challenging to draw definitive conclusions regarding the relative efficacy of the two regimens. Catapano et al. used both RTX dosing regimens to treat refractory SLE and, although not in a formal comparative setting, did not find significant differences in B cell depletion levels, clinical outcomes, or adverse effects (50). Therefore, the two-week doses of two 100 mg might be more convenient and could become the preferred treatment regimen for patients with SLE and LN.

In clinical practice, RTX is rarely used alone; it is often combined with glucocorticoids or with both glucocorticoids and immunosuppressants. When used in combination therapy, the dose of glucocorticoids is gradually reduced as clinical symptoms improve, significantly enhancing efficacy and reducing the risks of infection, bone marrow suppression, liver function impairment, and secondary malignancies (71, 72).

## Efficacy and safety of RTX

To date, extensive clinical research has been conducted on rituximab, and its efficacy is still uncertain. Both randomized controlled trials of rituximab for SLE patients, the EXPLORER and LUNAR studies, failed to meet their primary endpoints. In the EXPLORER study, 257 patients with moderate to severe non-renal SLE were randomly assigned to receive either RTX or placebo treatment. RTX was administered at a dose of 1,000 mg at weeks 0, 2, 24, and 26, against a background treatment of azathioprine (AZA), methotrexate (MTX), or mycophenolic acid (MPA). At week 52, there was no significant difference between the treatment group and the placebo group in terms of the primary endpoint (73). In the LUNAR study, 144 patients with class III or IV lupus nephritis (LN) receiving mycophenolate mofetil (MPA) treatment were randomly assigned to receive either a placebo or rituximab (RTX) treatment. Similarly, in this study, RTX failed to achieve the primary endpoint, and there was no significant difference between the placebo and treatment groups in the proportion of patients achieving complete or partial renal remission (59).

However, numerous clinical trials and case reports have observed significant efficacy and reliable safety of RTX in patients with SLE and LN (74). In the study by Yi et al., patients in the RTX group had lower 24-h urinary protein and SLEDAI scores and a significantly higher complete remission rate than those in the CTX group (75). Looney et al. concluded that RTX relieved symptoms in most patients with refractory severe SLE (76). Ramos et al. found that RTX significantly improved symptoms in 91% of patients with refractory and recurrent LN (77). In addition, several systematic evaluations and network meta-analyses have analyzed the efficacy and safety of RTX in the treatment of LN (78–80), suggesting that RTX has significant clinical efficacy and good safety, making it a promising therapy for the treatment of SLE and LN, particularly for refractory severe SLE and refractory LN. Jens Vikse et al. retrospectively analyzed the clinical data of 70 patients with systemic inflammatory and autoimmune diseases and treated with long-term rituximab (≥16 years). In their study, infections and persistent dysgammaglobulinemia were the most common adverse events, occurring in 34.3 and 25.7%, respectively. End organ damage occurred in two patients, and no opportunistic infections were observed. Three patients died of lethal infection during the observational period. They concluded that long-term rituximab treatment is relatively well tolerated, and that no cumulative side effects were observed (81). In 2012, the American College of Rheumatology recommended RTX as a second-line treatment for refractory class III and IV LN. Additionally, the Chinese guidelines for the diagnosis and treatment of lupus nephritis indicate that for refractory or frequently relapsing LN, RTX can be used in combination therapy (71, 72). Furthermore, the 2019 management recommendations for LN, jointly developed by the European League Against Rheumatism (EULAR) and the European Renal Association-European Dialysis and Transplant Association, proposed that for non-responsive and refractory LN, RTX can also be used either as monotherapy or as an adjunct to mycophenolate mofetil (MMF), mycophenolic acid (MPA), or cyclophosphamide (CYC) (13).

The clinical efficacy of rituximab in treating systemic lupus erythematosus (SLE) demonstrates considerable variability, probably attributable to the following factors: elevated levels of BAFF (B-cell activating factor), B-cell reconstitution and the disease-specific high degree of heterogeneity in lupus. B-cell reconstitution after the infusion of rituximab is associated with increased BAFF levels. Elevated BAFF promote autoreactive B-cell proliferation and survival (82). Additionally, SLE is a highly heterogeneous disease with various pathogenic mechanisms, including autoreactive B-cells, alterations in TLR receptor function, differences in the IFN-*α* pathway, T-cell dysfunction, etc. (83). This heterogeneity may result in diverse patient responses to treatment, suggesting that a single therapeutic approach may not address all underlying mechanisms.

To overcome these challenges, potential strategies include combination therapy and sequential treatment. The potential of combining anti-B cell and anti-BAFF therapies should be further explored. Besides, in terms of sequential treatment, clinical studies of belimumab administration followed by RTX or RTX administration followed by belimumab are currently under investigation (84, 85).

## Conclusion

As an anti-CD20 monoclonal antibody B cell depleting agent, rituximab (RTX) has shown promising efficacy in several retrospective and open-label studies. Despite this, continued monitoring of RTX’s significant biological effects is necessary to evaluate long-term clinical benefits and risks. Considering the pathogenic significance of the B cell family in SLE and LN, targeting B cells and plasma cells presents a highly attractive therapeutic approach for SLE and LN. Understanding and advancing B cell biology in SLE and LN is crucial. In addition to the specific targeting of B cell surface antigens by RTX, treatment failures in SLE and LN have also been noted. This has driven interest in alternative targets for B cell activation, such as B lymphocyte stimulator (BlyS) and B cell activating factor (BAFF), which are expected to become focal points for future research. Additionally, selective targeting of B cell therapies will play a pivotal role in the personalized treatment management of SLE and LN patients. Moving forward, further work is needed to elucidate the full potential of B cell depletion strategies through drugs like RTX in the clinical setting.
